# Temperature and Prey Availability Drive Seasonal Variations in Diet, Abundance and Condition of *Forsterygion lapillum* Across Two Coastal Habitats

**DOI:** 10.1002/ece3.73390

**Published:** 2026-04-09

**Authors:** Anna Carolina Resende, Catarina Vinagre, Alice Rogers

**Affiliations:** ^1^ School of Biological Sciences Victoria University of Wellington New Zealand Wellington New Zealand; ^2^ CCMAR ‐ Centre of Marine Sciences University of Algarve Faro Portugal; ^3^ Universidade Do Algarve Faro Portugal; ^4^ MARE – Marine and Environmental Sciences Centre, Universidade de Lisboa, Faculdade de Ciências Lisboa Portugal

**Keywords:** climate change, gut content, macro‐invertebrate community

## Abstract

Environmental fluctuations in coastal waters have been increasing due to climate change and are likely to affect marine fish physiology and behaviour. Increases in temperature and turbidity can compromise food intake, body condition and abundance, among other vital traits. Environmental variations may also shape macro‐invertebrate community composition, which can lead to changes in food availability and feeding selectivity, given that they are a common food source for higher trophic levels. Both ocean temperature and turbidity are known to vary considerably from season to season, and different populations of a species can be locally adapted to varying environmental conditions. To address gaps relating to the influence of environmental variability on coastal fish populations, this study examines how temperature, turbidity and prey availability influence the diet, body condition and abundance of *Forsterygion lapillum* throughout the year in two neighbouring coastal ecosystems. We found seasonal and spatial variation in *F. lapillum* body condition, abundance, total gut volume and diet. Moreover, we found that *F. lapillum* body condition, abundance and total gut volume were positively correlated with warmer temperatures, while no clear patterns relating to turbidity were found. The macro‐invertebrate community was found to differ between areas and seasons. Our results suggest that prey quality varies seasonally across both study areas, and the composition of macro‐invertebrate community seems to drive *F. lapillum* diet and prey selectivity in these natural populations.

## Graphical abstract

This study investigated how temperature, turbidity and prey availability affect the diet, body condition and abundance of *F. lapillum* in two coastal ecosystems. Results showed seasonal and spatial variation, with body condition, abundance and gut volume positively correlated with warmer temperatures but not with turbidity. Differences in macro‐invertebrate communities across sites and seasons were found to shape diet and feeding selectivity in *F. lapillum* .

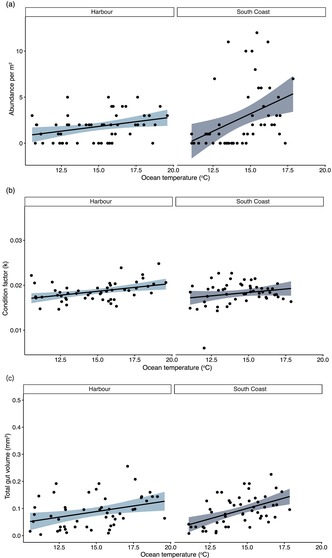

## 1 Introduction

Climate change is recognised as one of the most important global‐scale threats to marine ecosystems (Hoegh‐Guldberg and Bruno 2010; Tittensor et al. 2021; Guo et al. 2022). It is projected to alter coastal environments through chemical and physical changes, including increases in temperature (Guo et al. 2022), changes in salinity and pH (Du et al. 2019; Findlay and Turley 2021) and increases in turbidity (Roberts 2012; IPCC 2024). Marine organisms exhibit physiological and behavioural responses to rapid climate change (Demello and Phillips 2011; Pecl et al. 2014; Camp et al. 2018; Glockner‐Fagetti and Phillips 2020), yet responses to the combined effects of multiple stressors remain poorly understood (Burleson and Silva 2011; McBryan et al. 2013; Breitburg et al. 2018).

Temperature is an important abiotic factor that affects all organisms, and in particular ectotherms like marine macro‐invertebrates and fish. (Perry et al. 2005; Scott et al. 2019; Kreiling et al. 2021). Increases in ocean temperature influence processes from the individual to ecosystem scale, and include changes in behaviour (Chabot and Guénette 2013; Metcalfe et al. 2016; Smale et al. 2017), population dynamics (Atkinson et al. 2004; Poloczanska et al. 2016; Smale et al. 2017) and food web structure (O'Connor et al. 2009; Kortsch et al. 2019; Reum et al. 2024). In fish, behavioural and metabolic stress responses include alterations to food intake (Salin et al. 2016; Scott et al. 2017), changes in body condition (Kamimura et al. 2021; Lindmark et al. 2023), and compromised growth (Jiang et al. 2021; Canosa and Bertucci 2023). Additionally, changes in temperature alter macro‐invertebrate community composition (Figueroa et al. 2021; Kreiling et al. 2021). Macro‐invertebrates include benthic primary consumers (Cowles et al. 2009; Chen et al. 2021) that play key roles within food webs, and are an abundant and common food source for higher trophic levels, including reef‐associated fish (Wenger et al. 2018; Audzijonyte et al. 2023). A shift in macro‐invertebrate community composition is likely to influence or limit fish feeding behaviours (Kreiling et al. 2021; Kotalik et al. 2023).

Turbidity is another environmental variable that affects marine organisms (Seers and Shears 2015; Bass et al. 2023). The input or re‐suspension of fine sediment particles and other dissolved organic matter can increase turbidity in coastal environments (Smith and Schindler 2009; Seers and Shears 2015; Zanghi and Ioannou 2025). Increases in turbidity impact fish both directly and indirectly, for example by reducing aerobic performance (Rosewarne et al. 2016; Hess et al. 2017) or interfering with prey capture due to reduced visual acuity (Lowe et al. 2015, Hess et al. 2017, Wenger et al. 2018).

In intertidal coastal areas, increases in both temperature and turbidity often co‐occur, exposing fish to direct physiological stressors, but also indirect effects of changes in prey quality and availability (Henderson et al. 2021; Lindmark et al. 2023). Prey availability can influence prey selection of generalist predators (O'Gorman et al. 2016; Kreiling et al. 2021). For instance, when prey is scarce in the environment, a generalist predator may adopt one of two strategies: it might consume a wide range of different prey, regardless of quality, to reduce foraging time; or it may invest more effort in locating high‐quality prey, thereby maximising energy intake (Schoener 1971; Zango et al. 2019). Conversely, when prey is abundant, selectively targeting prey items with the highest energetic value may be more advantageous, hence a more selective feeding behaviour is expected (Emlen 1966; Kreiling et al. 2021).

Temperature, turbidity and prey availability (i.e., macro‐invertebrate community) are known to vary considerably from season to season (Greig et al. 1988; Seers and Shears 2015; Berlajolli et al. 2019). Seasonal fluctuations in coastal waters are increasing due to climate change and are likely to continue to do so into the future (Carton et al. 2015; Gupta et al. 2020), with consequences for fish ecology (Pörtner et al. 2014; Poloczanska et al. 2016). Moreover, fish respond physiologically and behaviourally to the characteristics of their local environment (Demello and Phillips 2011; Glockner‐Fagetti and Phillips 2020). Species that do not relocate in response to environmental change are expected to (a) adapt to local conditions through long‐term generational adaptation and/or (b) acclimatise to short‐term variations, such as fluctuations of biotic and abiotic conditions due to seasonality (Pörtner et al. 2014). Local adaptation and acclimatisation can reduce the vulnerability of populations to environmental change by forcing intraspecific behavioural and physiological differentiation, thereby enabling different populations to live in a wide range of heterogeneous environments (Kelly et al. 2012; Pörtner et al. 2014; Barros et al. 2022). Previous research has established that this is particularly observed in fish on small spatial scales (Hess et al. 2017; Willis et al. 2021; Paul et al. 2021), therefore, it is essential to examine how natural populations of the same fish species differ in their behavioural responses to environmental variation.

The common triplefin, *Forsterygion lapillum* (Hardy 1989), is an abundant benthic intertidal fish found throughout New Zealand (McDermott and Shima 2006). This species reaches a maximum reported length of 6.7 cm and a maximum recorded age of 3 years (Fricke 1994). *F. lapillum* inhabits a variety of heterogeneous environments, including harbours, coastal reefs and rock pools, and is typically found at depths from 0 to 20 m (Fricke 1994; McDermott and Shima 2006). It can tolerate a wide range of temperature fluctuations from winter to summer, making it an ideal model species for investigating responses to environmental variability (Hilton et al. 2010; Khan et al. 2014). Given a limited understanding of how spatial and seasonal variations in environmental conditions and prey availability influences fish ecology, this study addresses the following research questions:

1. How do spatial and seasonal differences affect *F. lapillum* abundance, condition, prey availability and diet?
2. How do fluctuations in environmental conditions (i.e., temperature and turbidity) affect the diet, condition and abundance of *F. lapillum* ?
3. How does prey availability affect *F. lapillum* diet and prey selectivity?

## 2 Methods

### 2.1 Study Area

**FIGURE 1:**
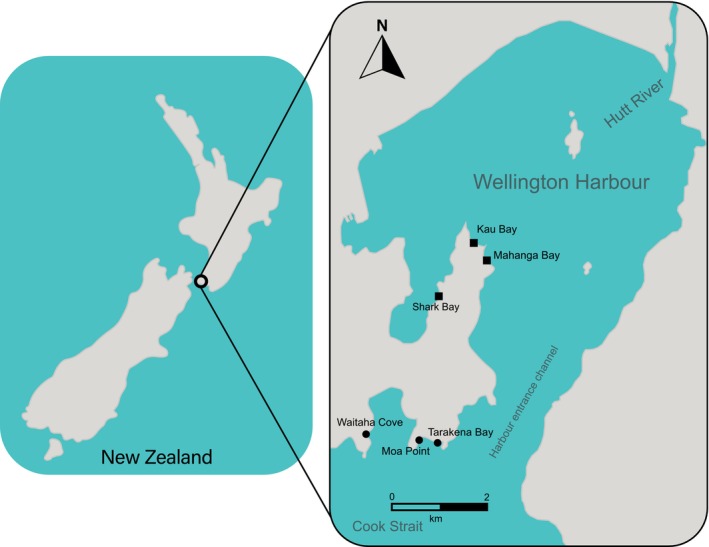
Map showing the location of each sampling site. Squares indicate harbour sites and circles indicate south coast sites.

The present study encompasses two distinct neighbouring areas along the coastline of Wellington, New Zealand, the Wellington Harbour and the Wellington South Coast. Three sites were studied in each area; Shark Bay (41°18′ 7.46″S, 174°49′3.05″ E), Kau Bay (41°17′16″ S, 174°49′48″ E) and Mahanga Bay (41°17′ 32″S, 174° 50′ 8″E) in the Wellington Harbour, and Moa Point (41°20′ 29″S, 174°48′ 40″E), Tarakena Bay (41°20′ 32″S, 174°49′ 10″E), and Waitaha Cove (41°20′ 27″S, 174°47′ 30″E) on the Wellington South Coast (Figure 1). The two study areas were chosen due to differences in physical conditions and anthropogenic use (Booth 1975; Glockner‐Fagetti and Phillips 2020), which drive differences in environmental conditions such as temperature regimes and sedimentation levels. The Wellington harbour is partially isolated from oceanic waters and experiences freshwater input from the Hutt River, with a catchment area of about 630 km^2^ (Booth 1975). The inner harbour has an average depth of 20 m and an average tide current speed of 0.10 m.s^−1^. It is considered to have moderate to extreme turbidity (Cassie 1960), with increases in sediment input after European settlement (Lewis and Mildenhall 1985), due to high shipping activity and river discharges (Brodie 1958; Maxwell 1956). Other anthropogenic impacts in the harbour include widespread marine debris (e.g., plastics, fishing gear), and substrate alteration from coastal development (Halpern et al. 2008; Micaroni et al. 2024).

By contrast, the Wellington South Coast is a highly exposed environment adjacent to the Cook Strait, characterised by large swells, frequent exposure to high‐energy southerly winds and storm events that affect the wave regime (Berman 2010; Glockner‐Fagetti and Phillips 2020). The average maximum wave height in the south coast is of 8 m and tide current speeds can fluctuate from 0.11 to 3.6 m.s^−1^, usually reaching an average of 2.5 m.s‐1 (Stanton et al. 2001; Berman 2010; Walters et al. 2010). The nearshore depths in the south coast vary from 0 to 30 m (Micaroni et al. 2024). As tide currents become progressively stronger, consistent motion of the bottom substrate can be observed and intertidal areas are subjected to periodic sediment re‐suspension events, and pulses in turbidity (Carter and Lewis 1995; Berman 2010). Fishing and anchoring represent common anthropogenic pressures along the Wellington South Coast (Micaroni et al. 2024).

### 2.2 Environmental Conditions

Environmental variables, namely temperature and sedimentation, were measured in both study areas from April 2023 to March 2024. Temperature was measured using HOBO TidbiT v2UTBI‐001 loggers, programmed to log water temperature hourly. Hourly measurements were averaged to provide weekly temperatures over the sampling period. The loggers were fixed to concrete blocks placed at 2 m depth and were retrieved and changed each season (Figure S1). Since sediment re‐suspension decreases light levels in coastal environments, turbidity values were used as a proxy for re‐suspended sediment and were measured biweekly at each site using the sensor RBRconcerto3 C.T.D, reported in nephelometric turbidity units (NTU).

### 2.3 Field Surveys and Sampling

*F. lapillum* abundance was quantified on snorkel in the Wellington Harbour and Wellington South Coast once per season (second month of the season) from April 2023 to March 2024. Abundance was assessed in 1 × 1 m (*n* = 15) quadrats that were placed haphazardly along three transects perpendicular to shore (10 m) and at least 20 m parallel to one another (Nagelkerken et al. 2018).

At each sampling site (*n* = 3 per study area), *F. lapillum* (*n* = 15) was collected once per season (second month of the season) from April 2023 to March 2024 for gut content analysis. Sampling was carried out using hand nets at an average depth of 1.2 m, at least 200 m away from where visual surveys were taken (Willis et al. 2021). Fish were euthanised by immersion in an ice slurry (Blessing et al. 2010) and then taken to the Coastal Ecology Laboratory (VUCEL), where they were weighed and measured before having their gastrointestinal (GI) tract removed, and preserved in 1.5 mL micro‐tubes with 70% ethanol. Only adult fish were quantified and sampled in both study areas. Average weight and length of fish collected were 1.55 ± 0.39 g and 4.36 ± 0.37 cm respectively for Wellington Harbour fish, and 2.11 ± 0.58 g and 4.72 ± 0.55 cm for Wellington South Coast fish, based on *F. lapillum* minimum adult size (Mensink and Shima 2014).

To assess the availability of common prey items, benthic light traps (*n* = 3) made from PVC tubes, with a funnel on one end and a cap in the other end holding a green light inside (Figure S2, modified from McLeod and Costello 2017), were placed haphazardly and left overnight at each site once per season (second month of the season) from April 2023 to March 2024 (Kelso et al. 2012). On the day following the light trap placement, macro‐invertebrates were removed from the light traps and preserved in 15 mL vials with 70% ethanol until processing.

### 2.4 Diet, Prey Availability and Condition Factor

GI content of *F. lapillum* was analysed under a stereomicroscope using two methods: (a) frequency of occurrence (%Fo), which is the number of times each item occurs as a percentage of the total number of occurrences of all items (Hynes 1950), and (b) volumetric (V), where each prey volume was measured on a Petri dish with a 4 mm square grid against a 1 mm reference height (Hellawell and Abel 1971), with a conversion to volume (1 mm^3^ = 0.001 mL) for each food item. The ingested items that could not be identified in fish guts due to advanced stages of digestion were classified as ‘non‐ID’.

Macro‐invertebrates found in light traps were also analysed using the volumetric method (Hyslop 1980), with abundance estimated based on the total volume to individual volume ratio. Food items and macro‐invertebrates were identified to the lowest taxonomic level possible. Other items that were not considered food but that appeared in GI were also quantified and named.

The body condition of *F. lapillum* was estimated using the Fulton condition factor *K*, calculated as:

$$K=\frac{100*W}{L^{3}}$$

where *W* is fish weight and *L* is fish length.

### 2.5 Statistical Analysis

All statistical analysis and graphic representations were conducted in R, version 4.2.2 (R Core Team 2022). Homogeneity test of multivariate dispersion (PERMDISP) was used to assess differences in *F. lapillum* diets and macro‐invertebrate prey communities between study areas and seasons. Using PERMDISP, the distance of the centroid was calculated through a principal co‐ordinate analysis (PCoA), using the Bray–Curtis dissimilarity index, allowing for the comparison of the average dissimilarity in individual observations within groups (Anderson 2006). To check for overall differences in *F. lapillum* diet and available prey between areas and seasons, we performed PERMANOVA tests with the *adonis2* function in the *vegan* package using the Bray–Curtis dissimilarity method with 999 permutations (Anderson, 2001; Oksanen et al. 2013), followed by a pairwise comparison between seasons performed with the *pairwiseAdonis* package (Martinez Arbizu 2017). An analysis of similarity percentages (SIMPER) (Clarke 1993) was used to calculate the contribution of each taxa to the observed patterns shown in the PERMDISP ordination for both the gut content macro‐invertebrate community data. Two‐way ANOVA tests were performed interacting the independent variables of area and season to investigate variations in *F. lapillum* abundance, total gut volume and condition factor. Abundance values were log transformed and condition factor values were Box‐Cox transformed using the MASS package to perform the ANOVA analysis (Venables and Ripley 2002). A *post hoc* comparison test and pairwise contrasts were performed with the *emmeans* package (Lenth, 2020), using the Tukey method. Abundance and condition factor are displayed as box plots and total gut volume is shown as mean and standard deviation in bar plots, all using the *ggplot2* package (Wickham, 2016). Pearson's correlation tests were used to examine the relationship between ocean temperature and turbidity on *F. lapillum* abundance, total gut volume and condition factor, performed and shown in line graphs using the package *ggpubr* (Kassambara, 2023). Prey preference was analysed using *econullnetr* package (Vaughan et al. 2017) through network‐based null models. Binary data indicating presence or absence of prey in guts were used alongside macro‐invertebrate community abundance data (i.e., prey availability) to calculate prey selectivity.

## 3 Results

### 3.1 Seasonal and Spatial Differences

**FIGURE 2:**
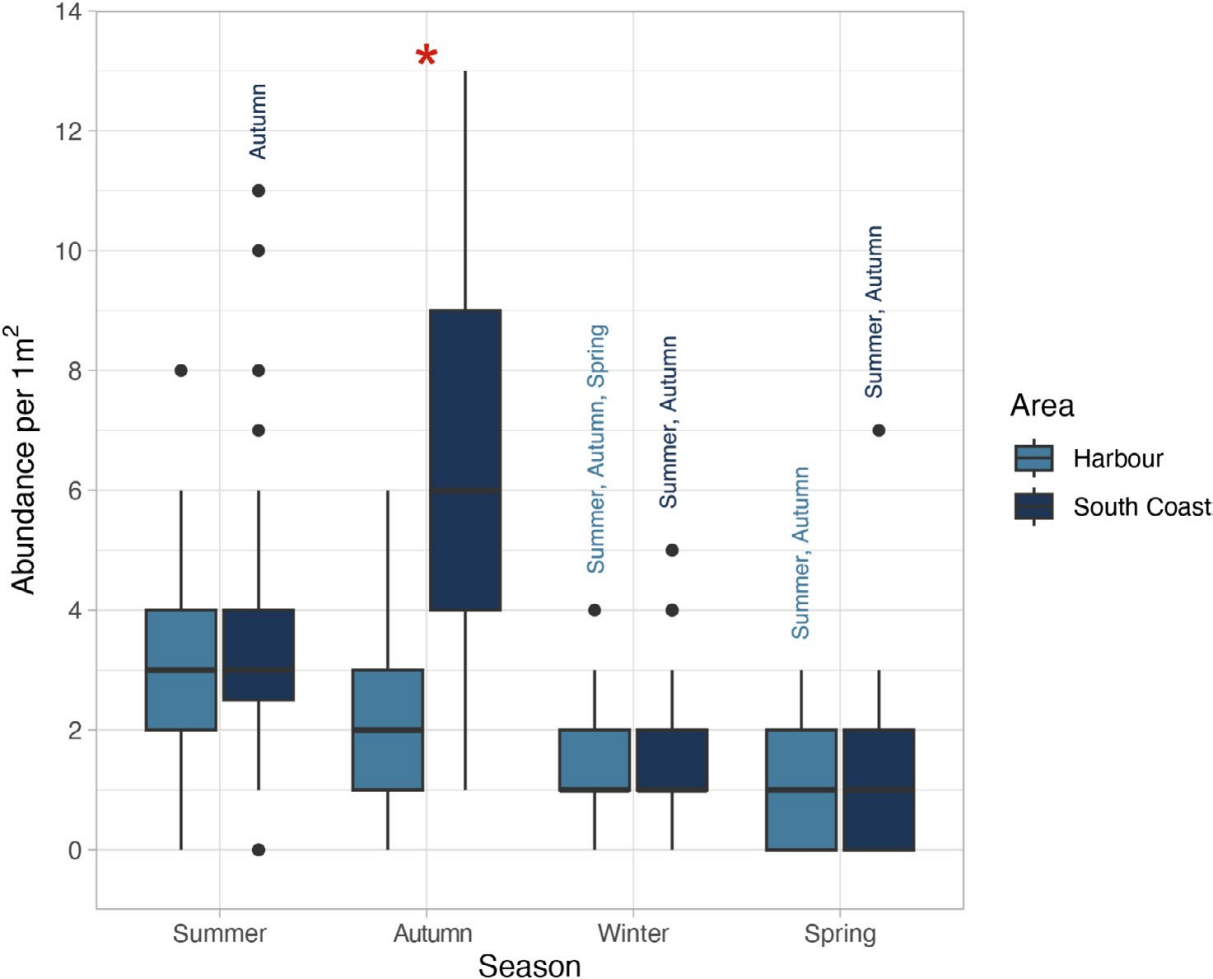
Box plot showing adult *F. lapillum* abundance per m^2^ in the Wellington harbour and south coast (*n* = 15 per area each season). Asterisks indicate significant differences between both areas (*p* < 0.05). Seasons names above the box plots represent differences between seasons within the same area.

The abundance of *F. lapillum* significantly differed between areas across seasons (*F*_(2.630)_ = 4.022, *r* ^2^ = 0.266, *p* = 0.007). Contrasts between areas revealed that abundance was about 2.75× higher in the Wellington South Coast than in the Wellington Harbour during autumn (*p* < 0.0001), with no variation between areas in other seasons (Figure 2).

Variations between seasons within areas were also found. In the Wellington Harbour, *F. lapillum* abundance was about 1.61× lower in winter than in summer (*p* = 0.0005) and autumn (*p* = 0.027), while 1.62× higher in winter than spring (*p* = 0.013). Additionally, abundance in spring was about 3× lower than in autumn (*p* < 0.0001) and summer (*p* < 0.0001).

On the Wellington South Coast, *F. lapillum* abundance was always higher in autumn, about 1.71× more than in summer (*p* = 0.0129), about 4× more than in winter (*p* < 0.0001) and about 5.46× more than in spring (*p* < 0.0001). Similarly, abundance was 2.35× greater in summer than in winter (*p* < 0.0001) and 3.16× higher than in spring (*p* < 0.0001).

**FIGURE 3:**
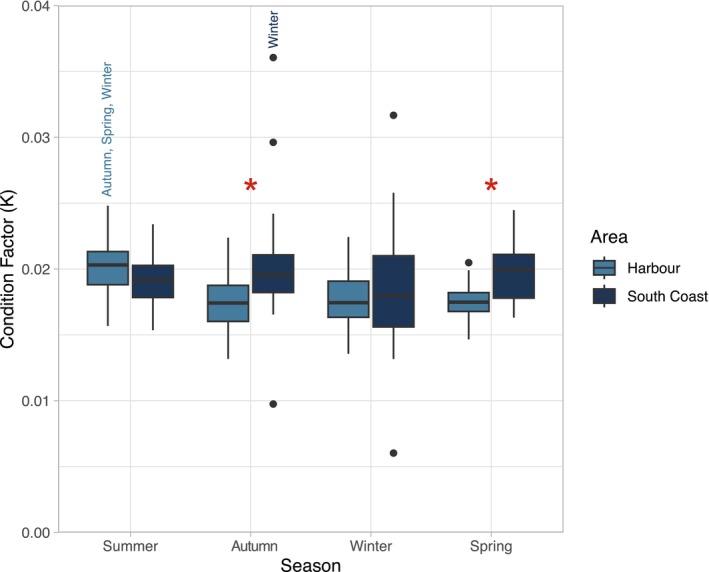
Box plot showing adult *F. lapillum* condition factor (K) in the Wellington harbour and south coast (*n* = 45 per area each season). Asterisks indicate significant differences between both areas (*p* < 0.05). Seasons names above the box plots represent differences between seasons within the same area.

*F. lapillum* body condition, determined by condition factor (K) differed between areas and across seasons (*F*_(2.635)_ = 6.074, *r* ^2^ = 0.113, *p* = 0.0005) (Figure 3). Comparing study areas revealed differences in condition between Wellington Harbour and Wellington South Coast in autumn (*p* < 0.0001) and spring (*p* = 0.0031), when fish from the Wellington South Coast always showed better body condition. For Wellington Harbour, body condition was always better during summer compared to autumn (*p* < 0.0001), winter (*p* = 0.0003) or spring (p = 0.0003). For Wellington South Coast, body condition only differed between autumn and winter (*p* = 0.0055), and was better during autumn.

**FIGURE 4:**
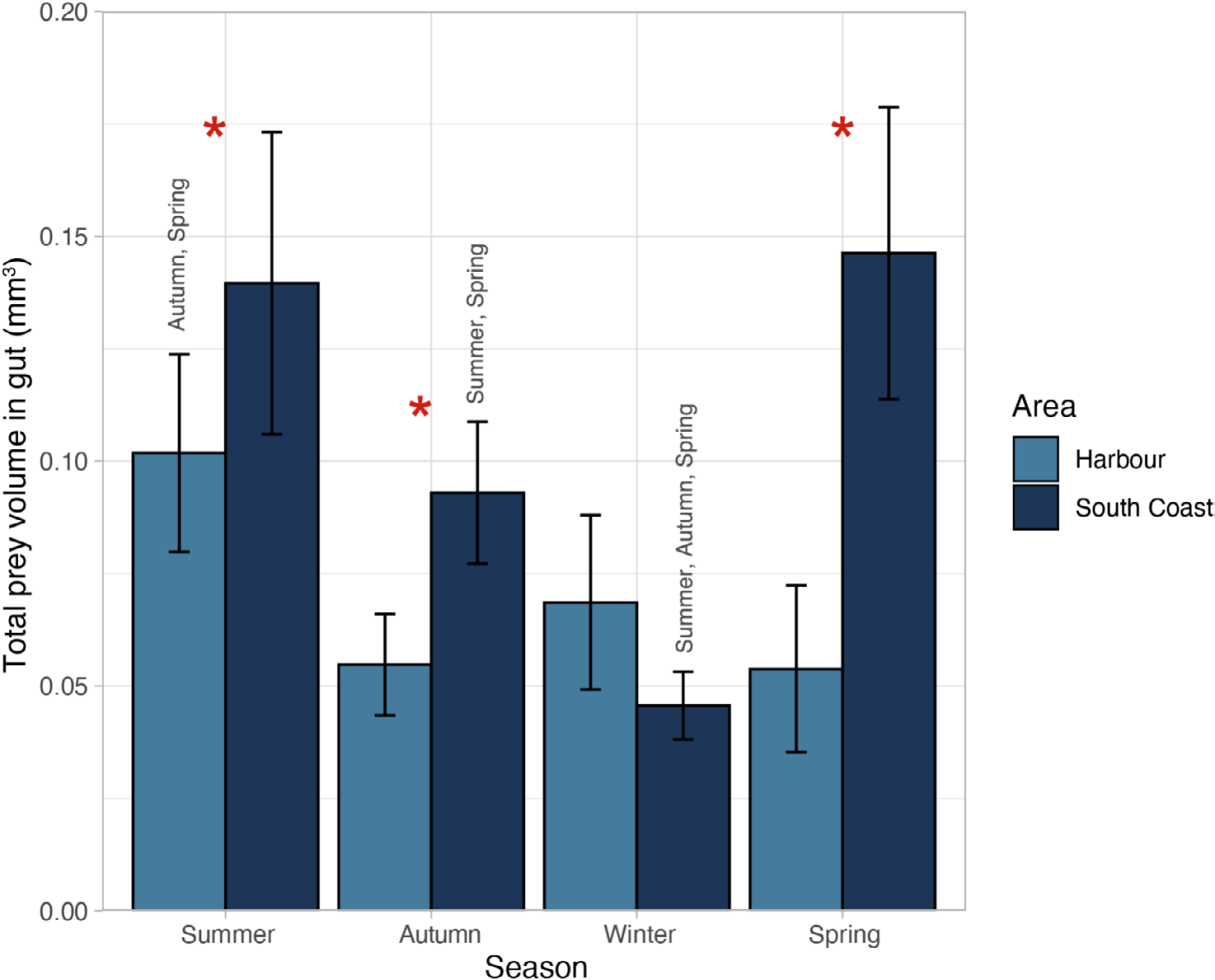
Bar plot showing total volume in gut of adult *F. lapillum* in the Wellington harbour and south coast (*n* = 45 per area each season). Asterisks indicate significant differences between both areas (*p* < 0.05). Seasons names above the box plots represent differences between seasons within the same area.

The total volume of prey and other items found in *F. lapillum* guts differed significantly across seasons and between areas (*F*_(2.636)_ = 12.07, *r* ^2^ = 0.264, *p* < 0.0001) (Figure 4). Fish from the Wellington Harbour and Wellington South Coast showed significant differences in total gut volume during summer (*p* = 0.0141), autumn (*p* = 0.0014) and spring (*p* < 0.0001), with highest volumes always found in fish guts from the Wellington South Coast. Seasonal differences in total gut volume were also observed within both study areas. In the Wellington Harbour, total gut volume was higher in summer in comparison to both autumn and spring (*p* = 0.0034). In the Wellington South Coast, total gut volume was higher in summer when compared to autumn (*p* = 0.0044) and winter (*p* < 0.0001). Similarly, total gut volume was also higher in spring in comparison to autumn (*p* = 0.0005) and winter (*p* < 0.0001). Collectively, these results indicate greater seasonal effects on total gut volume for the Wellington South Coast fish compared with the Wellington Harbour fish (Figure 4).

### 3.2 Environmental Variables

**FIGURE 5:**
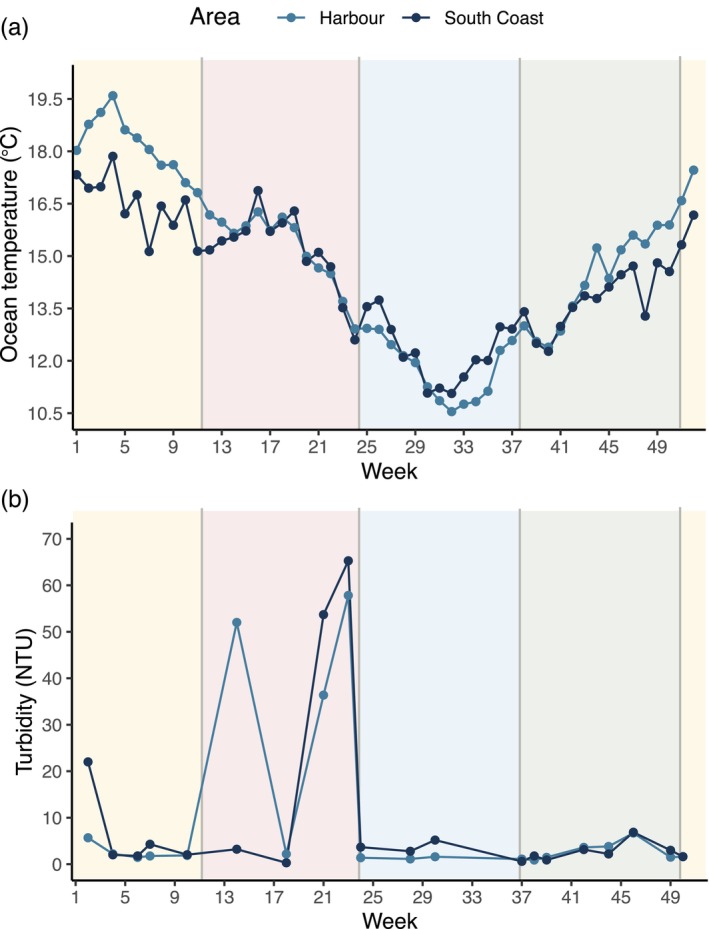
Seasonal variation of (a) ocean temperature (°C) and (b) turbidity (NTU) around the Wellington harbour and south coast. Lines connect averages calculated from biweekly (turbidity) or hourly averaged per week (ocean temperature) measurements taken from autumn 2023 until summer 2024 in three sites in the harbour and three sites in the south coast. Shaded regions denote different seasons: Summer (yellow), autumn (red), winter (blue) and spring (green).

The Wellington Harbour monthly mean temperature ranged from 10.5°C to 19.3°C, reaching a maximum of 21.4°C in summer and a minimum of 9.9°C in winter (Figure 5a). The Wellington South Coast monthly mean temperatures ranged between 10.9°C and 17.6°C, reaching a maximum of 19.4°C in summer and a minimum of 10.2°C in winter (Figure 5b). The Wellington Harbour experiences temperatures of about 2°C higher than the Wellington South Coast in spring and summer, while experiencing similar temperatures during autumn and winter. In both areas, turbidity ranged from 0 to ~200 NTU and reached the highest observed values during autumn in both areas. The turbidity and temperature data are used below as predictors of *F. lapillum* abundance, body condition and total gut volume.

**FIGURE 6:**
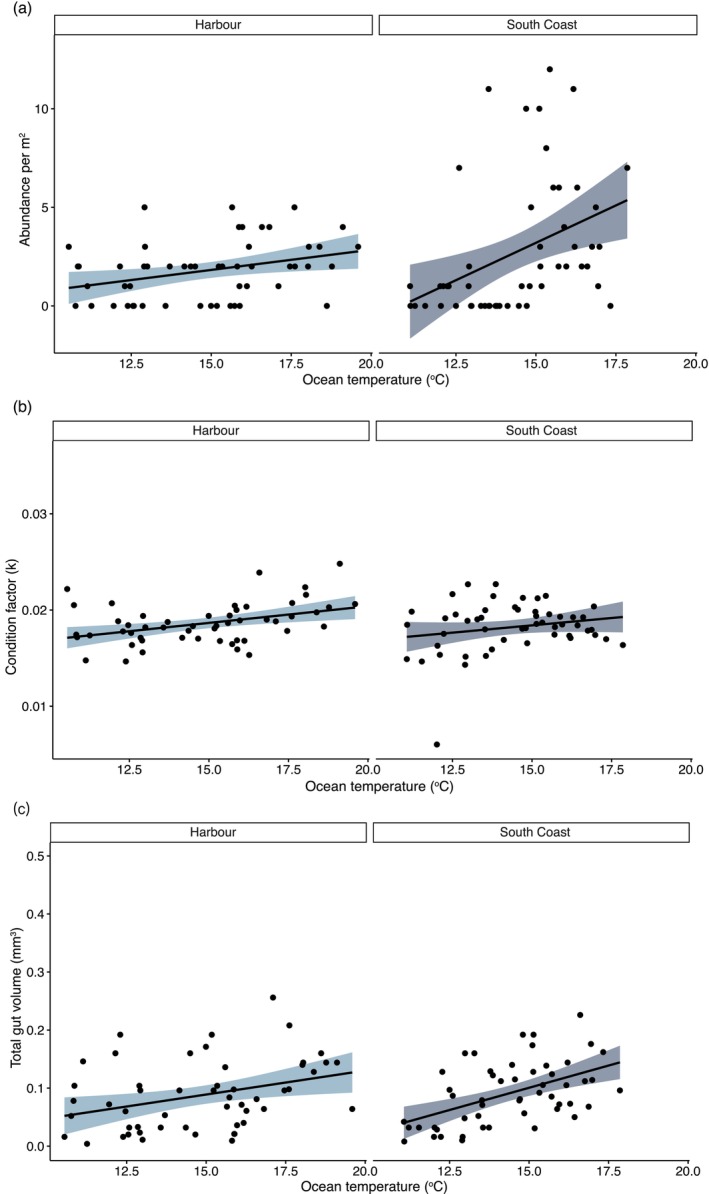
The correlation between (a) abundance, (b) condition factor (c) total gut volume and ocean temperature in the Wellington harbour and the south coast (*n* = 90 per area).

*F. lapillum* abundance (*r* ^2^ = 0.37, *p* = 0.0064), body condition (*r* ^2^ = 0.4, *p* = 0.003) and total gut volume (*r* ^2^ = 0.34, *p* = 0.013) in Wellington Harbour increased significantly with increases in ocean temperature (Figure 6a–c). In the Wellington South Coast, *F. lapillum* abundance (*r* ^2^ = 0.52, *p* < 0.0001) and total gut volume (*r* ^2^ = 0.51, *p* < 0.0001) also increased significantly with increases in ocean temperature, while body condition did not.

Conversely, variations in turbidity did not affect any of the assessed variables for both fish from the harbour and the south coast.

### 3.3 *F. lapillum* Diet

**FIGURE 7:**
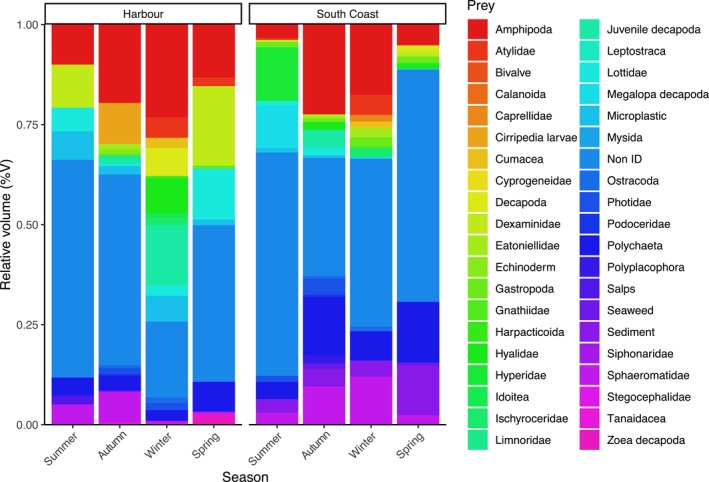
Relative volume of prey groups found in *F. lapillum* stomachs in each season from autumn of 2023 until summer of 2024, in the Wellington harbour (left) and Wellington south coast (right) (*n* = 9 per area each season). The Non ID group represents organic matter that was heavily digested and unidentifiable. Items that are not considered part of their diet but were found in their stomach, namely micro‐plastic, sediment, seaweed and salps, are also being shown.

A total of 40 distinct food items were identified in the guts of *F. lapillum* across all seasons, with gut content varying seasonally and between locations. In both the Wellington Harbour and the Wellington South Coast, food item diversity in *F. lapillum* guts was higher in autumn and winter and lower in summer. Patterns of gut content composition and overlap across seasons and study areas are presented in Figure 7.

Items classified as ‘non‐ID’ represented the highest volume found in *F. lapillum* guts in both areas, 39.98% of total gut volume across seasons for fish in the Wellington Harbour, and 46.29% in the Wellington South Coast. The highest prey volumes and occurrence prevalent in fish guts from Wellington Harbour were of Amphipoda, with the highest volume consisting of general Amphipoda for winter (23.16%) and autumn (19.57%) and Dexaminidae during spring (19.65%) and summer (10.66%) (Table 1, Figure 1). Similarly, the most frequent food item found in fish guts from the Wellington South Coast was Amphipoda in all seasons, with the family Hyperidae being the most frequent in summer (Table 1). Amphipoda also was the highest prey volume found in the Wellington South Coast in winter (17.63%) and autumn (22.43%), while Polychaeta (15.13%) and Hyperidae (13.42%) represented the second highest prey volume found in spring and summer, respectively (Figure 1). The least common food item found in *F. lapillum* guts was Leptostraca and Salpida, occurring in less than 5% of fish stomachs across all seasons and in both areas.

**TABLE 1:** Frequency of occurrence (Fo%) of food items in the stomachs of *F. lapillum* in both areas across. Values in bold represent the highest occurrence for each area per season.

		Harbour				Southcoast			
		Summer	Autumn	Winter	Spring	Summer	Autumn	Winter	Spring
Crustacea									
Amphipoda		**32.14**	**52.17**	**51.35**	**53.57**	18.51	**52.17**	**28.26**	**43.33**
	Atylidae	0	0	10.81	3.57	3.70	0	6.52	0
	Caprellidae	0	0	0	0	0	0	2.17	0
	Cyproideidae	0	0	0	0	0	0	2.17	0
	Dexaminidae	17.85	0	0	17.85	3.70	0	0	3.33
	Hyalidae	0	0	8.10	0	0	4.34	0	3.33
	Hyperidae	0	0	0	0	**33.33**	0	0	0
	Ischyroceridae	0	2.17	13.51	0	0	2.17	2.17	0
	Photidae	0	4.34	10.81	3.57	7.40	13.04	0	6.66
	Podoceridae	0	2.17	0	0	0	4.34	0	0
	Stegocephalidae	0	0	2.70	0	0	0	2.17	0
Cirripedia	Larvae	0	8.69	0	0	0	0	0	0
Copepoda									
	Calanoida	0	0	5.40	0	0	0	4.34	0
	Harpacticoida	0	0	5.40	0	0	0	2.17	0
Cumacea		0	0	5.40	0	0	0	2.17	3.33
Decapoda		0	0	5.40	0				3.33
	Juvenile	0	8.69	5.40	0	0	2.17	0	3.33
	Megalopa	0	0	0	0	3.70	0	2.17	0
	Zoea	0	0	0	7.14	0	0	0	0
Isopoda									
	Gnathiidae	3.57	0	2.70	3.57	0	0	6.52	0
	Idoitea	0	0	5.40	0	0	0	0	0
	Limnoridae	0	0	0	0	0	4.34	0	0
	Sphaeromatidae	14.28	8.69	5.40	7.14	7.40	26.08	21.73	6.66
Leptostraca		0	0	0	0	0	2.17	0	0
Mysida		0	2.17	0	0	0	0	0	0
Ostracoda		0	2.17	8.10	3.57	0	2.17	6.52	6.66
Tanaidacea		0	4.34	0	0	0	2.17	0	0
Echinoderma		0	8.69	0	0	0	2.17	0	0
Mollusca									
Bivalve		7.14	0	0	14.28	7.40	0	0	20
Gastropoda		0	4.34	0	0	11.11	21.73	17.39	0
	Eatoniellidae	0	13.04	0	7.14	7.40	21.73	15.21	26.66
	Lottidae	10.71	2.17	8.10	10.71	11.11	6.52	2.17	3.33
	Siphonariidae	0	0	0	0	0	0	0	0
Polyplacophora		0	0	0	0	0	4.34	0	0
Polychaeta		3.57	6.52	5.4	10.71	11.11	21.73	10.86	13.33
Salpida		3.57	0	0	0	0	0	0	0
Seaweed		0	0	0	0	0	4.34	0	3.33
Debris		0	0	0	0	0	0	0	0
	Non ID	82.14	71.73	51.35	75	88.88	58.69	80.43	93.33
	Microplastic	21.42	26.08	18.91	14.28	18.51	10.86	0	3.33
	Sediment	0	6.52	0	0	18.51	19.56	13.04	50

**FIGURE 8:**
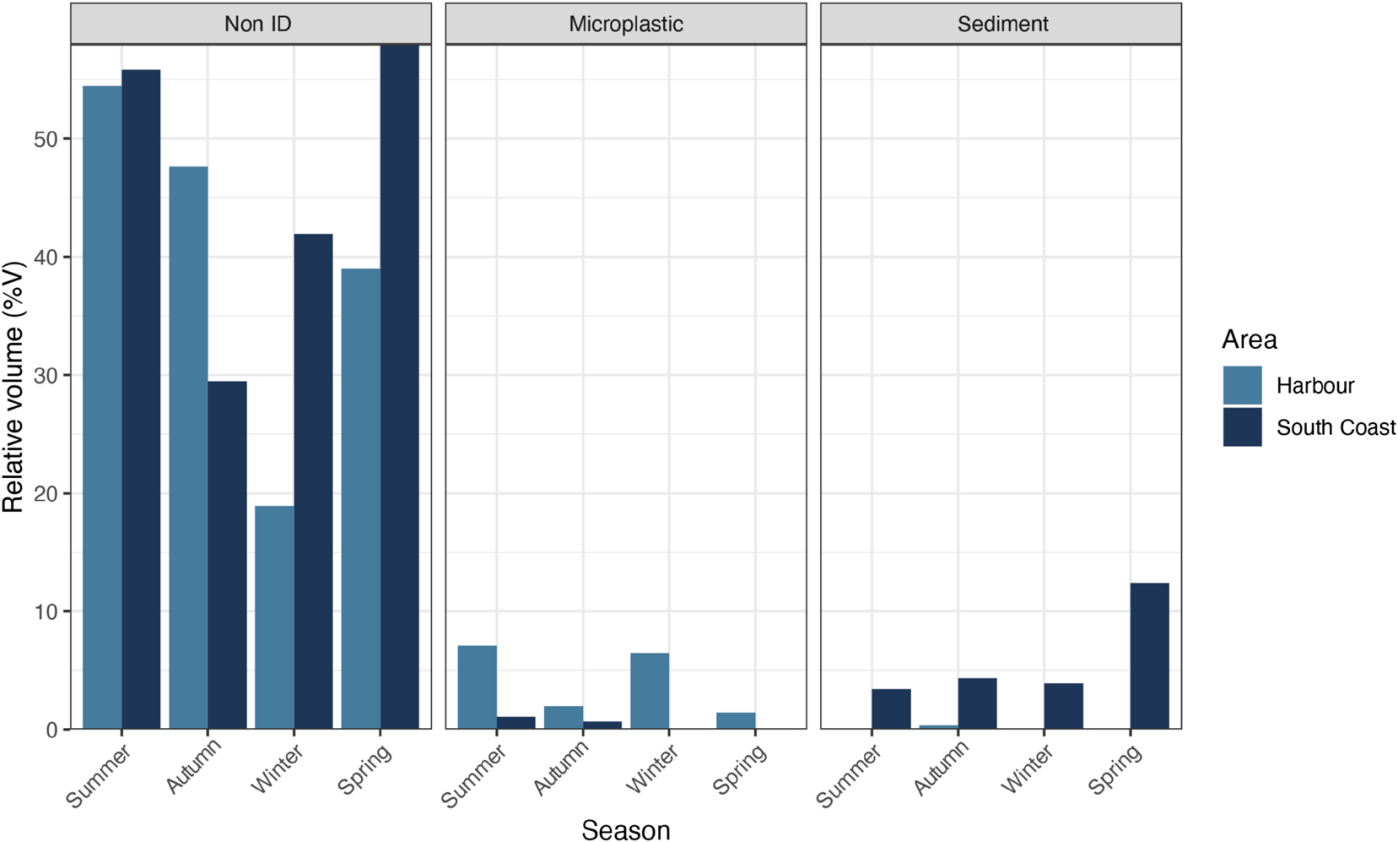
Relative volume of items that are not prey found in *F. lapillum* stomachs, highlighting seasonal differences in the Wellington harbour and the south coast (*n* = 9 per area each season).

Non‐organic items, such as micro‐plastics and sediments, were also found in fish guts. Micro‐plastics were found in both areas across almost all seasons, except for winter in the Wellington South Coast. Sediment was found in all fish guts from the Wellington South Coast, but only found in Wellington Harbour fish during autumn. Sediment was classified as debris and grouped with non‐ID in Table 1 and Figure 8.

**FIGURE 9:**
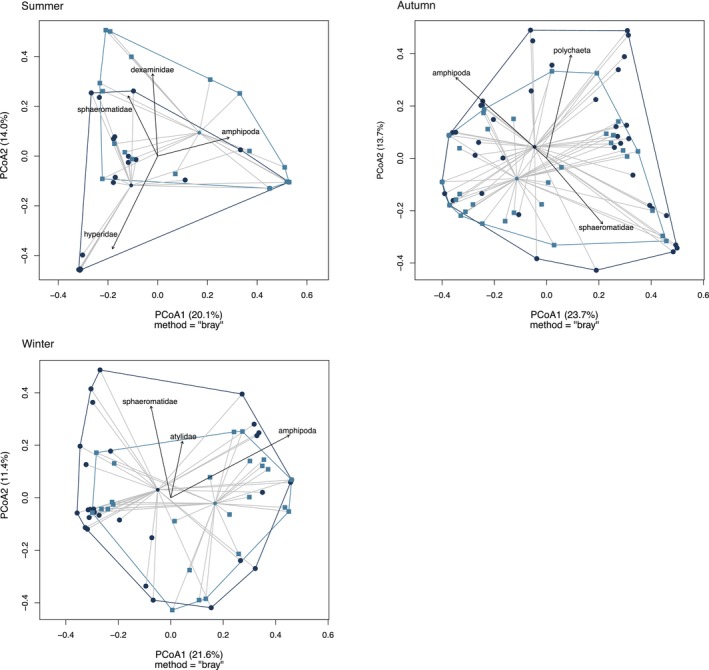
PCoA of *F. lapillum* gut content variation comparing harbour and south coast per season (*n* = 45 per area each season). Dark blue represents south coast and light blue represents harbour. The hulls were marked around the distance between each individual and the group median (centroid). Length and direction of the vectors indicate strength of association between the ordination and associated labelled taxa.

Significant differences in gut content between study areas were found in the summer (PERMANOVA, pseudo‐*F* = 2.340, *R* ^2^ = 0.0610, *p* = 0.004), autumn (PERMANOVA, pseudo‐F = 2.333, R^2^ = 0.0305, *p* = 0.017) and winter (PERMANOVA, pseudo‐F = 2.738, R^2^ = 0.0450, *p* = 0.005), with no differences detected during spring. According to a SIMPER analysis, the most frequent food items in summer highlighting contrast between areas were Amphipoda (22.8%) and Dexaminidae (14.1%) for Wellington Harbour fish and Hyperidae (17%) and Sphaeromatidae (11.9%) for Wellington South Coast fish. In autumn, SIMPER analysis revealed that food groups contributing to the difference between areas were Amphipoda (32.6%) for Wellington Harbour fish, while for Wellington South Coast fish, Sphaerometidae contributed in 15.5% and Polychaeta contributed in 15.8%. In winter, SIMPER analysis showed that Amphipoda (29.2%) and Atylidae (9.4%) for Wellington Harbour fish contributed for differences in *F. lapillum* gut content between areas, while Sphaeromatidae contributed 9.4% to Wellington South Coast fish. PERMIDISP ordering with seasonal differences between areas are demonstrated in Figure 9.

A second ordering produced by PERMDISP showed little to no variation in gut content according to the food items in each of the sampled areas individually across seasons (Figure S4), which was confirmed by pairwise comparisons, with no significant differences for fish in Wellington Harbour. In Wellington South Coast fish, the only significant difference was found between autumn and summer (PERMANOVA, pseudo‐F = 3.95, R^2^ = 0.062, *p* = 0.006). A pairwise SIMPER analysis showed that the most frequent food items distinguishing autumn and summer in the Wellington South Coast were Amphipoda, Hyperidae, Polychaeta and Sphaeromatidae, which together accounted for 67.6% of the variation.

### 3.4 Prey Availability

A total of 56 macro‐invertebrate groups were found in light traps, 45 in Wellington Harbour and 50 in Wellington South Coast, with an overlap of 42 groups (across all seasons) found in both areas. Amphipoda were by far the most abundant group in all seasons and areas, reaching a maximum of 8724 individuals in spring in the Wellington South Coast and a minimum of 322 in Wellington Harbour in winter. The second most abundant group found in Wellington Harbour was Mysida, with a maximum relative abundance of 1186 in spring. In Wellington South Coast, the second most abundant group was Decapoda in the zoea phase, reaching a relative maximum of 1207 in spring (Table 2).

**TABLE 2:** Macro‐invertebrates community composition sampled across seasons in both assessed areas. Values in bold represent the highest occurrence for each area per season.

		Harbour				South coast			
		Summer	Autumn	Winter	Spring	Summer	Autumn	Winter	Spring
Crustacea									
Amphipoda		**1794**	1135	322	**3608**	**7402**	**5123**	**1871**	**8724**
	Amphilochidae	0	4	32	0	0	0	17	10
	Ampithoidae	4	0	3	0	7	0	0	1
	Atylidae	1	6	116	5	13	26	45	148
	Caprellidae	2	0	0	1	1	1	1	0
	Cyproideidae	0	5	8	0	8	4	50	0
	Dexaminidae	26	70	92	157	641	66	30	21
	Eophliantidae	27	23	322	33	219	28	17	247
	Hyalidae	100	11	55	84	130	226	7	136
	Hyperidae	4	0	0	1	89	0	4	7
	Lysianassidae	16	3	29	25	312	23	40	214
	Ischyroceridae	18	4	1	33	179	157	19	107
	Melphidippidae	339	0	69	480	207	0	0	0
	Oedicerotidae	0	0	0	0	11	0	0	5
	Phliantidae	15	0	0	2	22	16	17	17
	Photidae	2	4	8	4	16	15	16	23
	Phoxocephalidae	1	0	6	9	135	1	1	142
	Podoceridae	2	0	3	1	3	11	1	0
	Pontogeneiidae	230	2	62	356	447	198	146	399
	Stegocephalidae	0	1	4	0	5	110	7	33
	Stenothoidae	2	3	0	0	2	26	4	16
	Tryphosidae	0	0	0	0	29	5	7	3
Cirripedia	Larvae	0	**2500**	0	0	0	0	0	0
Copepoda									
	Calanoida	485	194	95	111	10	1	0	30
	Harpacticoida	0	2	60	0	0	5	2	2
	Peltidae	772	52	62	30	39	67	16	52
	Siphonotomastoida	0	24	0	0	0	0	0	0
Cumacea		19	32	7	18	127	532	255	291
Decapoda		2	0	0	0	0	0	0	0
	Juvenile	23	16	0	12	1	5	0	196
	Megalopa	97	1	0	54	2	2	0	77
	Zoea	108	40	4	385	119	44	6	1075
Caridae		7	23	51	20	1	20	4	0
Isopoda									
	Amphoroidea	57	19	0	7	65	15	1	33
	Cirolanidae	0	0	0	0	72	9	0	193
	Gnathiidae	154	32	24	90	35	364	158	122
	Hemioniscidae	1	0	0	0	22	15	2	50
	Idoitea	2	2	0	3	15	19	0	5
	Limnoridae	0	0	0	0	11	1	0	2
	Sphaeromatidae	32	0	43	19	352	103	155	384
Leptostraca		0	0	0	0	6	1	0	3
Mysida		932	751	**1091**	1186	118	27	1	22
	Larvae	14	0	0	2	20	0	0	0
Ostracoda		105	27	8	85	340	226	27	130
Tanaidacea		10	30	0	2	29	3	7	5
Tetrasquillidae		2	0	0	0	3	0	0	1
Echinoderma		0	0	0	0	0	13	0	0
Mollusca									
Cephalopoda		13	0	0	0	2	19	3	0
Gastropoda		0	0	20	6	7	1	10	9
	Eatoniellidae	0	0	0	0	0	9	0	8
Polychaeta		114	2	1	5	44	9	39	42
Salpida		17	0	0	0	0	0	0	0
Other									
	Eggs	0	0	0	0	518	0	0	0
	Fish larvae	0	0	0	3	6	0	0	5

**FIGURE 10:**
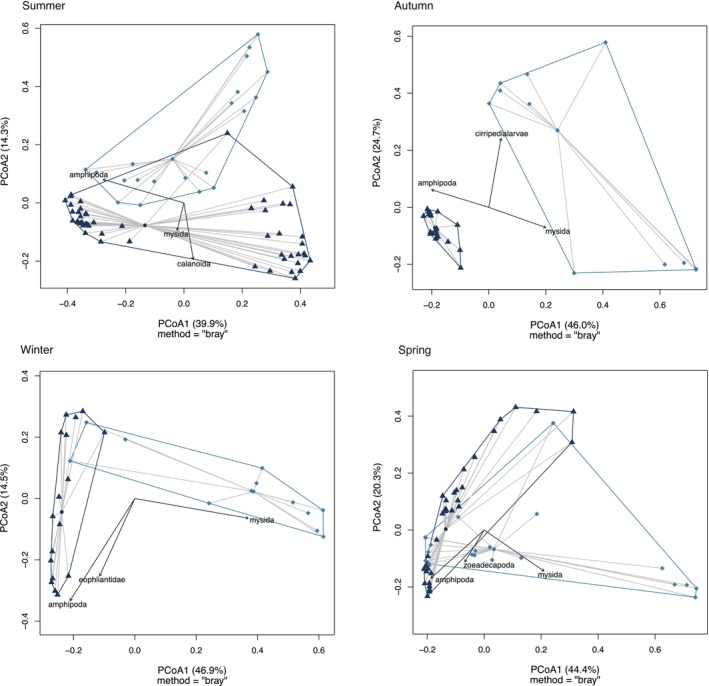
PCoA of macro‐invertebrate composition comparing harbour and south coast samples per season (*n* = 9 per area each season). Dark blue represents south coast and light blue represents harbour. The hulls were marked around the distance between each individual and the group median (centroid). Length and direction of the vectors indicate strength of association between the ordination and associated labelled taxa.

The macro‐invertebrate community composition (i.e., prey availability) differed between study areas and across seasons (PERMANOVA, *p* = 0.001). According to a SIMPER analysis, the main drivers of differences between study areas in all seasons was of Amphipoda in the Wellington South Coast and Mysida in Wellington Harbour (Figure 10).

### 3.5 Prey Selectivity

**FIGURE 11:**
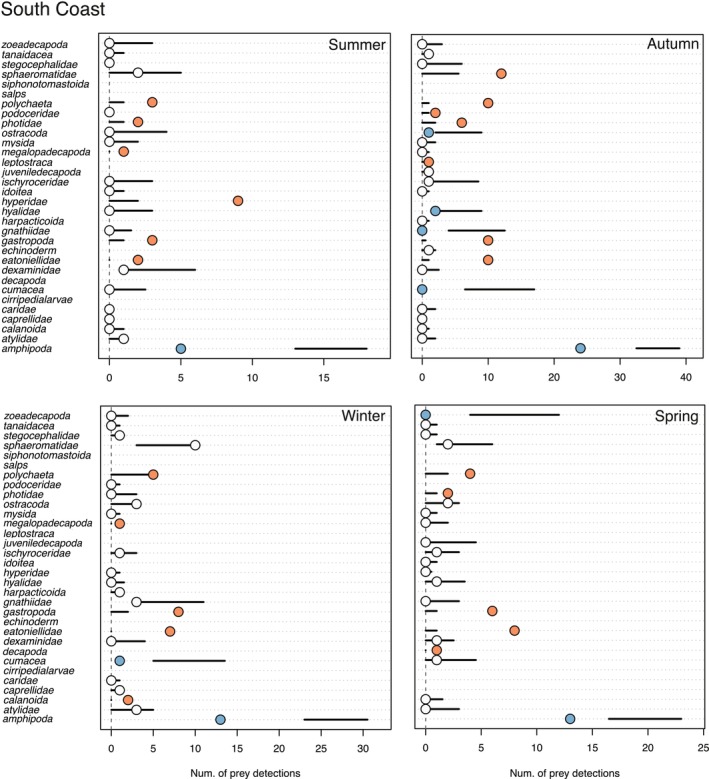
Prey preference or avoidance (i.e., difference between expected and observed feeding) of *F. lapillum* during different seasons in the South Coast. Horizontal lines denote 95% confidence limits of the expected frequency of interaction according to null models. Circles represent the observed frequency of predation. Blue = lower consumption than expected (avoidance or inaccessibility), white = as expected (in proportion to prey relative abundance), red = higher than expected (consumed more frequently than predicted from prey relative abundance).

**FIGURE 12:**
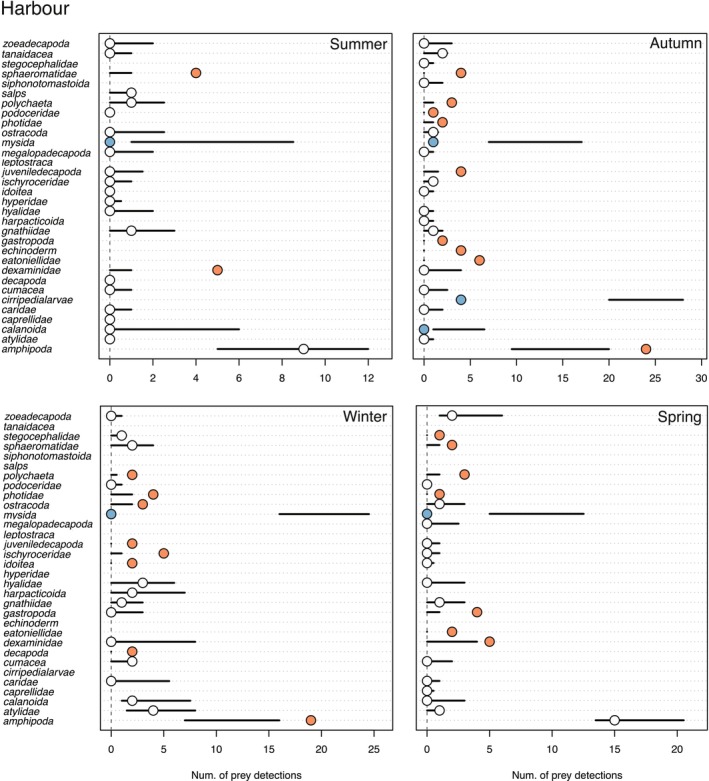
Prey preference or avoidance (i.e., difference between expected and observed feeding) of *F. lapillum* during different seasons in the Harbour. Horizontal lines denote 95% confidence limits of the expected frequency of interaction according to null models. Circles represent the observed frequency of predation. Blue = lower consumption than expected (avoidance or inaccessibility), white = as expected (in proportion to prey relative abundance), red = higher than expected (consumed more frequently than predicted from prey relative abundance).

*F. lapillum* from Wellington Harbour and the Wellington South Coast exhibited preferences or avoidance of specific taxa as determined by macro‐invertebrate communities (i.e., prey availability) in light traps and gut content samples in every season (Tables S1 and S2). Fish from the Wellington South Coast ate a higher than expected number of Gastropoda, Polychaeta and Eatoniellidae across all seasons, while eating a lower than expected number of Amphipoda (Figure 11). In autumn and winter, *F. lapillum* from the Wellington Harbour ate a higher than expected number of Amphipoda, while eating a higher than expected number of Sphaeromatidae in summer and spring. In addition, fish from the Wellington Harbour ate a lower than expected number of Mysida across all seasons (Figure 12).

## 4 Discussion

### 4.1 Seasonal and Spatial Differences

Understanding how seasonal and spatial differences affect natural fish populations is critically important, given that seasonal temperature ranges continue to increase due to climate change, and fish response can vary on small spatial scales (Da Silva et al. 2019; Willis et al. 2021).

*F. lapillum* exhibits high site fidelity (Shima et al. 2012), and in this study, we found that *F. lapillum* abundance was significantly higher during autumn in the Wellington South Coast in comparison to the Wellington Harbour. Autumn season coincides with the end of recruitment season for *F. lapillum* (Shima et al. 2012; Mensink and Shima 2014), and in a study analysing co‐occurrence between age classes of the common triplefin, Mensink and Shima (2014) found that the peak of adult density coincides with peak juvenile densities, which could indicate that there is a higher recruitment of *F. lapillum* in the Wellington South Coast in comparison to the Wellington Harbour. Moreover, fish abundance is highly dependent on prey availability (Johnson et al. 2012; Korman et al. 2021; Huang et al. 2021). When assessing the effect of prey abundance and size on the distribution of demersal fish, Johnson et al. (2012) found that fish abundance was higher in areas of high prey abundance. Accordingly, we found that *F. lapillum* abundance in autumn was higher in the Wellington South Coast, where the available prey is more diverse than in the Wellington Harbour. Similarly, when assessing how changes in prey availability affect a population of *Oncorhynchus mykiss* , Korman et al. (2021) found that there was a clear seasonal influence on prey availability, which ultimately affected fish survival and abundance across seasons.

*F. lapillum* abundance was lowest during spring in both study areas. A possible explanation for this is that spring (September–November) is *F. lapillum* breeding season (Moginie and Shima 2018), and throughout this period, males tend to be highly territorial and will remain site‐attached to defend their small cobbled breeding territories, only leaving occasionally for short‐distance foraging (Wellenreuther and Clements 2007; Moginie and Shima 2018).

Additionally, during autumn and spring, *F. lapillum* condition was better in the Wellington South Coast in comparison to the Wellington Harbour. A better fish condition indicates a positive growth in mass, which is facilitated by the quality of available resources and optimum feeding conditions (Stevenson and Woods 2006; Resende, Severo‐Neto, and Carvalho 2022). When assessing how fish condition was affected by changes in prey availability and density of competitors, Hiddink et al. (2016) found that the condition of benthivorous fish was positively related to the biomass of their prey. Equally important, in another study assessing how fish condition is linked to prey availability, Karlson et al. (2020) found that decreases in condition were linked to decreases in lipid‐rich prey. Hence, the higher condition factor of *F. lapillum* observed in most seasons on the South Coast may be attributed to the greater abundance and quality of available prey. Moreover, poor adult condition has been linked to decreases in fish survival rate and immediate effects on population abundance (Budy et al. 2007; Carim et al. 2017). In this context, the lower condition of *F. lapillum* from the Wellington Harbour warrants consideration as a possible factor affecting future population abundance.

### 4.2 Environmental Fluctuation Effects

Fluctuations in environmental conditions (e.g., temperature and turbidity) are known to affect fish abundance, condition and food intake (O'Gorman et al. 2016; Araújo et al. 2018; Kreiling et al. 2021; Lindmark et al. 2023). In the present study, *F. lapillum* abundance, condition and total gut volume were almost always positively correlated with increasing temperatures. In a thermal plasticity study with *F. lapillum* , Khan and Herbert (2012) found that regardless of what temperature acclimation fish were put through (15, 18, 21, 24 or 25°C), they preferentially selected temperatures of about 21°C. In laboratory studies, fish usually select temperatures at which their physiological performance is optimal, while in natural environments, the optimal temperature is also shaped by factors such as food availability and habitat quality (Khan and Herbert 2012; Payne et al. 2016). Optimal temperature regulates fish capacity to perform essential functions such as foraging and growth by maximising metabolic and physiological performance (Pörtner and Gutt 2016; Jonsson 2023). Given that not only *F. lapillum* abundance and condition, but also gut volume increased as temperatures in our study areas increased, we suggest that individuals in these natural populations are operating near their thermal optimum. This likely reflects enhanced foraging efficiency and overall physiological performance as the temperature in both study areas approached 21°C (maximum of 19.4°C in the Wellington South Coast and 21.4°C in the Wellington Harbour). It is important to note, however, that this positive correlation does not indicate that *F. lapillum* performance will continue to increase as temperature increases. In an acute stress study assessing ATP dynamics, Willis et al. (2021) found that *F. lapillum* might display reduced performance in temperatures above 25°C. Comparably, in the previously mentioned study by Khan and Herbert 2012, 55% mortality was observed in *F. lapillum* kept at 25°C after 2 weeks. Therefore, maximum temperatures beyond those currently observed in this study may exceed the thermal limits of *F. lapillum* , potentially leading to performance decline or increased mortality.

Increases in turbidity can affect fish foraging efficiency by either (i) reducing visual cues for predators that rely on visibility to forage or (ii) favouring species that rely on chemosensory mechanisms for predation (Parsons et al. 2014; Lunt and Smee 2015; Campbell and Taylor 2024). In the present study, we found no correlation between turbidity fluctuations and *F. lapillum* abundance, condition and total gut volume. Nevertheless, it is important to acknowledge that in this study it was only possible to measure turbidity biweekly. Given that ocean turbidity can fluctuate a lot on very short time‐scales in intertidal areas and change rapidly due to tidal currents (Seers and Shears 2015; Campbell and Taylor 2024), further studies assessing how highly dynamic habitats affect natural populations of *F. lapillum* on a shorter time‐scale are necessary.

### 4.3 Diet and Prey Selectivity

The triplefins around New Zealand have previously been classified as generalist omnivores, presenting a large diet breadth in accordance with habitat and prey size (Feary et al. 2009). In the present study, the predominant prey found in *F. lapillum* guts was composed of mobile benthic invertebrates, namely Amphipoda, Sphaeromatidae and Polychaeta. These macro‐invertebrates are rich in fatty acids and lipids (Prato et al. 2012; Hedberg et al. 2023). Given that lipids are the main source of energy for fish (Resende, Mauro Carneiro Pereira, et al. 2022; Hedberg et al. 2023), the presence of these food groups is likely adding quality to the diet of *F. lapillum* . Similarly, in a study on food‐web structure, Karlson et al. (2020) identified Amphipoda, Isopoda and Polychaeta as key dietary components for *Clupea harengus* and *Gadus morhua* , indicating their importance as a high‐energy source for fish in the studied area. During foraging, the selection of prey is likely influenced by the availability of suitable prey groups in the surrounding environment (Gill 2003). At temperate latitudes, seasonal changes in environmental conditions strongly affect macro‐invertebrate community composition and the input of high‐quality food, which have implications for higher trophic levels (Kreiling et al. 2021; Hedberg et al. 2023). Likewise, the prey availability and diet composition of *F. lapillum* differed among locations across seasons. In a trophic ecology study with two triplefin species, Polanco‐Pérez et al. (2021) found that intraspecific diet composition also varied in accordance with location, likely reflecting microhabitat differences and variation in prey availability. Although Mysida were the most abundant prey taxa available in Wellington Harbour, Amphipoda were the most frequent prey item found in *F. lapillum* guts. Previous research has described that optimal feeding strategies tend to be a trade‐off between resource quality, distribution and abundance (Emlen 1966; Polanco‐Pérez et al. 2021). For example, when prey availability is high, generalist predators can choose to eat either (a) high‐quality prey over more abundant prey or (b) mixed‐quality prey encountered, in order to spend less time and energy during foraging (O'Gorman et al. 2016; Kreiling et al. 2021). Whilst Mysida are also a lipid‐rich resource (Richoux et al. 2005), they are essentially pelagic invertebrates (Oliveira et al. 2023). Therefore, it is possible that in Wellington Harbour, *F. lapillum* is selecting benthic prey due to ease of capture, to reduce energy costs and minimise time spent foraging (Johnson et al. 2012).

Prey quality can be determined by prey size, palatability and ease of capture (Johnson et al. 2012). Previous studies have found that as the abundance of prey increases, so does the size selectivity of a predator (Gill 2003; Johnson et al. 2012). While Amphipoda were the most common prey available in the South Coast in all seasons, *F. lapillum* fed mostly on Polychaeta during spring. Additionally, the prey groups that highlighted differences between both assessed areas were Polychaeta and Sphaeromatidae. Previous studies show that the size selectivity of a predator increases as the abundance of available prey increases, given that energy gains may be optimal when bigger prey sizes are consumed (Johnson et al. 2012; Huang et al. 2021). Given that *F. lapillum* from the Wellington South Coast highly relied on bigger prey while foraging (Figure S3), we suggest that *F. lapillum* is likely to select prey based on size rather than abundance.

It is noteworthy that a major factor affecting prey size selectivity for a predator is the amount of food in a predator's stomach (e.g., fullness) (Gill 2003). In a study modulating prey size choice for frillfin goby, Tomida et al. (2012) have found that fish with empty stomachs were more motivated to search for prey while fish with partially full stomachs predominantly attacked the optimum prey size. In the present study, the total gut volume in *F. lapillum* guts was higher for Wellington South Coast fish during most seasons in comparison to Wellington Harbour fish. As a consequence, *F. lapillum* in the Wellington South Coast potentially spend less time foraging, with more resting periods between foraging bouts, favouring energy gains.

Our findings show that *F. lapillum* ingested very low amounts of Salpida, seaweed, micro‐plastic and sediment. We suggest that these items were inadvertently ingested by fish during foraging bouts (Li et al. 2021). As a cautionary note, we would like to note that the highest volume of content found in fish stomachs was represented by unidentifiable prey (e.g., highly digested items labelled as ‘non‐ID’). The presence of unidentifiable prey introduces uncertainty in determining the main prey consumed. However, this is a common challenge in most dietary studies in fish (Buckland et al. 2017), due to the difficulty in controlling all factors that influence prey digestion.

## 5 Conclusions

Intertidal fish experience large fluctuations in daily and seasonal temperature and therefore are expected to have the capacity to thermally acclimate to local variations in environmental conditions.

Seasonal and spatial variations in prey availability strongly influenced the diet, condition and abundance of *F. lapillum* , supporting their characterisation as generalist benthivorous carnivores.

Water temperature strongly predicts the distribution, condition and foraging behaviour of ectotherms, given that organisms perform better within their optimum thermal range. Our findings suggest that *F. lapillum* populations in the Wellington region may be living near their thermal optimum, as indicated by improved condition and foraging patterns at warmer temperatures. However, further increases in temperature may lead to performance decline or increased mortality, potentially exceeding *F. lapillum* thermal limits. Moreover, we found that *F. lapillum* behaviour was not affected by biweekly turbidity variations. Nonetheless, we acknowledge that behavioural and ecological shifts may not be captured in broader temporal scales, highlighting the importance of short‐term investigations.

*F. lapillum* diet was found to be flexible, with prey selectivity shaped by prey abundance and size. Furthermore, *F. lapillum* inhabiting areas with more diverse and bigger prey are likely to have better performance in comparison to those in areas with less quality of prey. Together, these results underscore the need for continued, fine‐scale research into environmental fluctuation effects on fish ecology, particularly as climate and coastal conditions continue to change.

## Author Contributions

**Anna Carolina Resende:** conceptualization (lead), formal analysis (lead), writing – original draft (lead), writing – review and editing (equal). **Catarina Vinagre:** writing – review and editing (equal). **Alice Rogers:** conceptualization (supporting), writing – review and editing (equal).

## Funding

This work was supported by Fundação para a Ciência e a Tecnologia, LA/P/0101/2020, UIDB/04326/2020, UIDP/04326/2020.

## Ethics Statement

We state that all fish collections and experimental designs were made in compliance with the special permit 711‐4 issued by the Ministry for Primary Industries of New Zealand and the project was approved by the Animal Ethics Committee of Victoria Wellington University, application number 0000030063.

## Conflicts of Interest

The authors declare no conflicts of interest.

## Supporting information


**Figure S1:** Photo of concrete block deployed at each sampling site, used to attach temperature loggers to measure temperature through seasons. Photo taken by recreational diver Paterson.
**Figure S2:** Photo of the costume made light trap deployed at each sampling site, used to sample macro‐invertebrates once per season. Photo taken by Matteo Colina.
**Figure S3:** Bar plot displaying average size of macro‐invertebrate prey found in light traps deployed at each sampling site.
**Figure S4:** PCoA of *F. lapillum* gut content variation in the south coast across seasons. The ellipses were marked in 95% C.I. Length and direction of the vectors indicate strength of association between the ordination and associated labelled taxa.
**Table S1:** Summary table for prey selectivity of *F. lapillum* during different seasons in the Harbour. Results from the test.interaction function in *econetnullr*.
**Table S2:** Summary table for prey selectivity of *F. lapillum* during different seasons in the South Coast. Results from the test.interaction function in *econetnullr*.

## Data Availability

The data supporting the findings of this study is publicly available on the Figshare repository in the following link. https://figshare.com/s/198939dab03ba3727b46.
