# Supplementary material for: Temperature and Prey Availability Drive Seasonal Variations in Diet, Abundance and Condition of Forsterygion lapillum Across Two Coastal Habitats
Source: Ecol Evol. 2026 Apr 9;16(4):e73390. doi: 10.1002/ece3.73390 (PMC13065880; doi:10.1002/ece3.73390)
Supplement: Supplementary file 1 — Figure S1: Photo of concrete block deployed at each sampling site, used to attach temperature loggers to measure temperature through seasons. Photo taken by recreational diver Paterson. Figure S2: Photo of the costume made light trap deployed at each sampling site, used to sample macro‐invertebrates once per season. Photo taken by Matteo Colina. Figure S3: Bar plot displaying average size of macro‐invertebrate prey found in light traps deployed at each sampling site. Figure S4: PCoA of F. lapillum gut content variation in the south coast across seasons. The ellipses were marked in 95% C.I. Length and direction of the vectors indicate strength of association between the ordination and associated labelled taxa. Table S1: Summary table for prey selectivity of F. lapillum during different seasons in the Harbour. Results from the test.interaction function in econetnullr. Table S2: Summary table for prey selectivity of F. lapillum during different seasons in the South Coast. Results from the test.interaction function in econetnullr. [file ECE3-16-e73390-s001.docx]

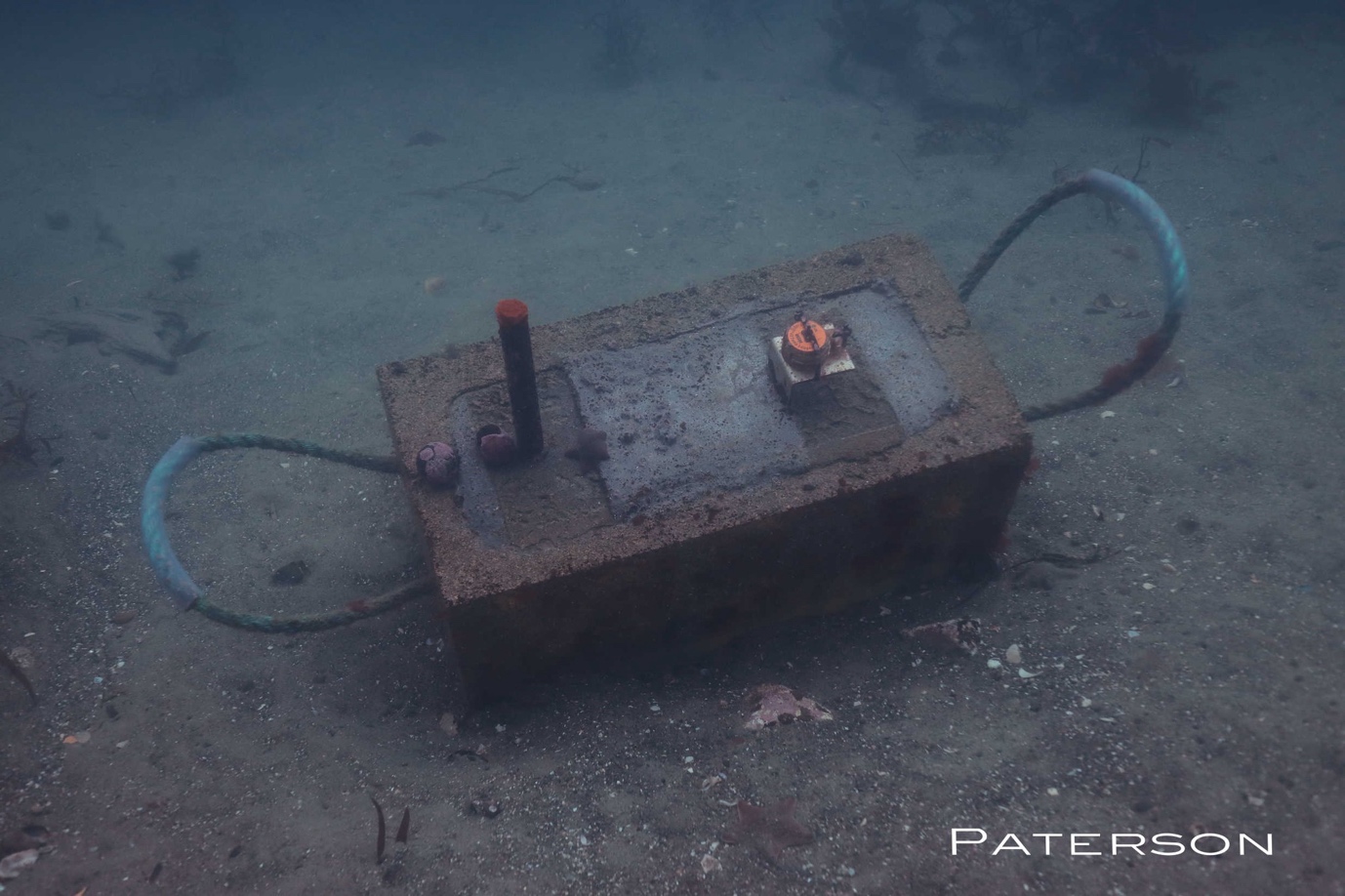


Figure S1 – Photo of concrete block deployed at each sampling site, used to attach temperature loggers to measure temperature through seasons. Photo taken by recreational diver Paterson.


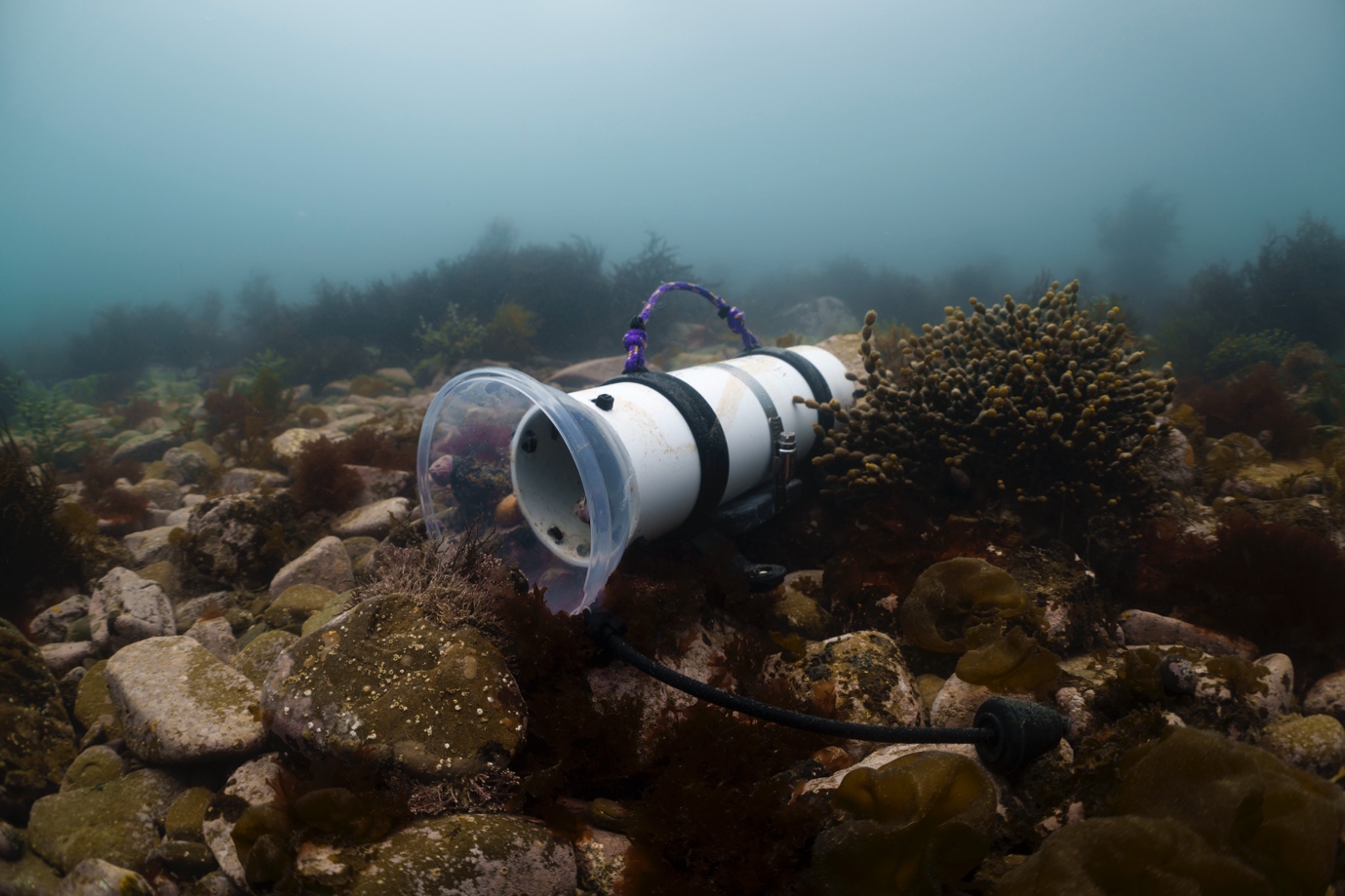


Figure S2 – Photo of the costume made light trap deployed at each sampling site, used to sample macroinvertebrates once per season. Photo taken by Matteo Colina.


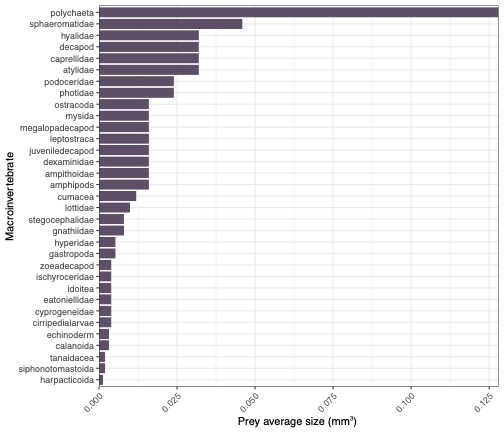
Figure S3 – Bar plot displaying average size of macroinvertebrate prey found in light traps deployed at each sampling site.

Figure S4 – PCoA of F. lapillum gut content variation in the south coast across seasons. The ellipses were marked in 95% C.I. Length and direction of the vectors indicate strength of association between the ordination and associated labelled taxa.

Table S1 – Summary table for prey selectivity of *F. lapillum* during different seasons in the Harbour. Results from the test.interaction function in *econetnullr*.

| Season | Resource | Observed | Null | Lower.95.CL | Upper.95.CL | Test |
| --- | --- | --- | --- | --- | --- | --- |
| Autumn | Amphipods | 24 | 14.95 | 9.475 | 20 | Stronger |
|  | Calanoida | 0 | 3.54 | 1 | 6.525 | Weaker |
|  | Cirripedialarvae | 4 | 23.93 | 20 | 28 | Weaker |
|  | Eatoniellidae | 6 | 0 | 0 | 0 | Stronger |
|  | Echinoderm | 4 | 0 | 0 | 0 | Stronger |
|  | Gastropoda | 2 | 0 | 0 | 0 | Stronger |
|  | Juveniledecapod | 4 | 0.28 | 0 | 1.525 | Stronger |
|  | Mysida | 1 | 11.45 | 7 | 17.05 | Weaker |
|  | Photidae | 2 | 0.08 | 0 | 1 | Stronger |
|  | Podoceridae | 1 | 0 | 0 | 0 | Stronger |
|  | Polychaeta | 3 | 0.08 | 0 | 1 | Stronger |
|  | Sphaeromatidae | 4 | 0 | 0 | 0 | Stronger |
| Summer | Dexaminidae | 5 | 0.14 | 0 | 1 | Stronger |
|  | Mysida | 0 | 4.55 | 1 | 8.525 | Weaker |
|  | Sphaeromatidae | 4 | 0.21 | 0 | 1 | Stronger |
| Winter | Amphipods | 19 | 10.8 | 7 | 16 | Stronger |
|  | Decapod | 2 | 0 | 0 | 0 | Stronger |
|  | Idoitea | 2 | 0 | 0 | 0 | Stronger |
|  | Ischyroceridae | 5 | 0.06 | 0 | 1 | Stronger |
|  | Juveniledecapod | 2 | 0 | 0 | 0 | Stronger |
|  | Mysida | 0 | 20.62 | 16 | 24.525 | Weaker |
|  | Ostracoda | 3 | 0.34 | 0 | 2 | Stronger |
|  | Photidae | 4 | 0.39 | 0 | 2 | Stronger |
|  | Polychaeta | 2 | 0.03 | 0 | 0.525 | Stronger |
| Spring | Dexaminidae | 5 | 2.09 | 0 | 4 | Stronger |
|  | Eatoniellidae | 2 | 0 | 0 | 0 | Stronger |
|  | Gastropoda | 4 | 0.08 | 0 | 1 | Stronger |
|  | Mysida | 0 | 9.25 | 5 | 12.525 | Weaker |
|  | Photidae | 1 | 0.02 | 0 | 0 | Stronger |
|  | Polychaeta | 3 | 0.05 | 0 | 1 | Stronger |
|  | Sphaeromatidae | 2 | 0.2 | 0 | 1 | Stronger |
|  | Stegocephalidae | 1 | 0 | 0 | 0 | Stronger |

Table S2 – Summary table for prey selectivity of *F. lapillum* during different seasons in the South Coast. Results from the test.interaction function in *econetnullr*.

| Consumer | Resource | Observed | Null | Lower.95.CL | Upper.95.CL | Test |
| --- | --- | --- | --- | --- | --- | --- |
| Autumn | Amphipods | 24 | 36.12 | 32.475 | 39 | Weaker |
|  | Cumacea | 0 | 11.52 | 6.475 | 17 | Weaker |
|  | Eatoniellidae | 10 | 0.25 | 0 | 1 | Stronger |
|  | Gastropoda | 10 | 0.03 | 0 | 0.525 | Stronger |
|  | Gnathiidae | 0 | 8.48 | 4 | 12.525 | Weaker |
|  | Hyalidae | 2 | 5.39 | 2.475 | 9 | Weaker |
|  | Leptostraca | 1 | 0.03 | 0 | 0.525 | Stronger |
|  | Ostracoda | 1 | 5.44 | 2 | 9 | Weaker |
|  | Photidae | 6 | 0.46 | 0 | 2 | Stronger |
|  | Podoceridae | 2 | 0.32 | 0 | 1 | Stronger |
|  | Polychaeta | 10 | 0.23 | 0 | 1 | Stronger |
|  | Sphaeromatidae | 12 | 2.59 | 0 | 5.525 | Stronger |
| Summer | Amphipods | 5 | 15.63 | 13 | 18 | Weaker |
|  | Eatoniellidae | 2 | 0 | 0 | 0 | Stronger |
|  | Gastropoda | 3 | 0.07 | 0 | 1 | Stronger |
|  | Hyperidae | 9 | 0.48 | 0 | 2 | Stronger |
|  | Megalopadecapod | 1 | 0.01 | 0 | 0 | Stronger |
|  | Photidae | 2 | 0.08 | 0 | 1 | Stronger |
|  | Polychaeta | 3 | 0.29 | 0 | 1 | Stronger |
| Winter | Amphipods | 13 | 27.01 | 23 | 30.525 | Weaker |
|  | Calanoida | 2 | 0 | 0 | 0 | Stronger |
|  | Cumacea | 1 | 9.47 | 5 | 13.525 | Weaker |
|  | Eatoniellidae | 7 | 0 | 0 | 0 | Stronger |
|  | Gastropoda | 8 | 0.45 | 0 | 2 | Stronger |
|  | Megalopadecapod | 1 | 0 | 0 | 0 | Stronger |
|  | Polychaeta | 5 | 1.86 | 0 | 4.525 | Stronger |
| Spring | Amphipods | 13 | 19.96 | 16.475 | 23 | Weaker |
|  | Decapod | 1 | 0 | 0 | 0 | Stronger |
|  | Eatoniellidae | 8 | 0.04 | 0 | 1 | Stronger |
|  | Gastropoda | 6 | 0.06 | 0 | 1 | Stronger |
|  | Photidae | 2 | 0.15 | 0 | 1 | Stronger |
|  | Polychaeta | 4 | 0.34 | 0 | 2 | Stronger |
|  | Zoeadecapod | 0 | 7 | 4 | 12 | Weaker |
